# Bacterial vaginosis and other infections in pregnant women in Senegal

**DOI:** 10.1186/s12879-021-06767-4

**Published:** 2021-10-23

**Authors:** Marion Bonneton, Bich-Tram Huynh, Abdoulaye Seck, Raymond Bercion, Fatoumata Diene Sarr, Elisabeth Delarocque-Astagneau, Muriel Vray

**Affiliations:** 1 Epidemiology of Infectious Diseases Unit, Institut Pasteur de Dakar, Dakar, Senegal; 2 Pharmacoepidemiology and Infectious Diseases Unit, Institut Pasteur, Paris, France; 3 Experimental Bacteriology Unit, Institut Pasteur de Dakar, Dakar, Senegal; 4 Faculty of Medicine, Pharmacy and Odontology, Cheikh Anta Diop University, Dakar, Senegal; 5 Medical Biology Laboratory, Institut Pasteur de Dakar, Dakar, Senegal; 6 Inserm, Paris, France; 7 Epidemiology of Emerging Diseases Unit, Institut Pasteur, Paris, France

**Keywords:** Bacterial vaginosis, Vaginal microbiome, Pregnant women

## Abstract

**Background:**

Bacterial vaginosis (BV) is associated with a higher risk of preterm delivery and spontaneous abortion. Yet little data on BV prevalence exist for sub-Saharan countries. The aim of this study was to estimate the prevalence of bacterial vaginosis and associated risk factors among pregnant women in Senegal.

**Methods:**

From October 2013 to December 2018, pregnant women in their third trimester were recruited in two primary health centers (one suburban, one rural) in Senegal. Healthcare workers interviewed women and collected a lower vaginal swab and a blood sample. Vaginal flora were classified into four categories using vaginal smear microscopic examination and Gram’s coloration. In our study, BV was defined as vaginal flora with no *Lactobacillus *spp. Variables associated with BV were analyzed using STATA® through univariate and multivariate analysis.

**Results:**

A total of 457 women provided a vaginal sample for analysis. Overall, BV prevalence was 18.6% (85/457) [95% CI 15.4–22.6]) and was similar in suburban and rural areas (18.9% versus 18.1%, p = 0.843). Multivariate analysis showed that primigravidity was the only factor independently associated with a lower risk of BV (aOR 0.35 [95% CI 0.17–0.72]).

**Conclusions:**

Our study showed significant BV prevalence among pregnant women in Senegal. Although the literature has underscored the potential consequences of BV for obstetric outcomes, data are scarce on BV prevalence in sub-Saharan African countries. Before authorities consider systematic BV screening for pregnant women, a larger study would be useful in documenting prevalence, risk factors and the impact of BV on pregnancy outcomes.

## Background

Adopted by the United Nations in 2016, the third sustainable development goal (SDG) is to “Ensure healthy lives and promote well-being for all at all ages.” Indeed, in 2015, neonatal mortality in Senegal was 47.2 per 1000 live births. According to the World Health Organization (WHO), 25% of neonatal deaths in Africa are caused by genital tract infections [1].

Bacterial vaginosis (BV) is defined as an imbalance of normal vaginal flora; it is characterized by high species diversity, depleted *Lactobacillus* spp., and increased anaerobes, such as *Gardnerella vaginalis*, *Atopobium vaginae* and other fastidious BV-associated bacteria [2]. BV symptoms include vaginal discharge and pruritus, although most women are asymptomatic [2]. Among pregnant women, however, BV is a risk factor of adverse obstetric outcomes [3, 4]. According to a meta-analysis conducted among 20,232 pregnant women [3], women with BV have two times the risk of preterm delivery, and nine time higher risk of spontaneous abortion. BV is also associated with a higher risk of sexually transmitted infections (STI) as Herpes Simplex Virus type 2 [5], Human Immunodeficiency Virus (HIV) [6–9], *Trichomonas vaginalis, Chlamydia trachomatis*, *Neisseria gonorrhea* [10] and Human Papilloma Virus (HPV) [11]. Bacterial vaginosis and low lactobacilli vaginal flora are associated with a delayed clearance of HPV and thus with increased risk of cervical intraepithelial neoplasia [12–15].

BV prevalence among sub-Saharan African countries varies from 25 to 50% [16] and from 9 to 23% in pregnant women [4, 17, 18]. In Senegal, one prospective study among non-pregnant women with symptoms of genital infections estimated a rate of 39.5% [19].

The aim of this study was to estimate the prevalence of bacterial vaginosis and associated factors among pregnant women in Senegal.

## Methods

### Data source, inclusion criteria and study setting

BIRDY (*Bacterial Infections* and antibiotic Resistant Diseases among Young children) is a multi-center cohort study launched to address the lack of epidemiological data concerning drug-resistant neonatal and infantile bacterial infections in three low-income countries (Cambodia, Madagascar and Senegal) [20]. Nested within the BIRDY study, the present study consecutively recruited pregnant women in Guédiawaye (suburban neighborhood in Dakar) and Sokone (rural area near the Gambian border) primary health centers during their third trimester of pregnancy, from October 2013 to September 2018. In the BIRDY study, healthcare workers interviewed women using a standardized questionnaire and collected from them a vaginal swab for streptococcus B (GBS). In addition, in our study, we also screened women for hepatitis B, *Toxoplasma gondii* and Rubella on blood sample and for vaginal candidiasis and BV on vaginal swab. All samples were transported in coolers to Pasteur Institute of Dakar laboratory within 24 h of collection, then stored in refrigerators until processed. Healthcare workers (nurses and midwives) were trained for this study.

### Data collection

Collected variables were as follows: (i) sociodemographic factors: age, marital status, education status (“formal education” was defined as women with at least primary education), sanitation type (indoor latrines and latrines with flushing water were considered to be improved sanitation facilities, whereas outdoor latrines without flushing water were designated unimproved sanitation facilities [21]); (ii) active smoking; (iii) nutritional status (estimated by mid-upper arm circumference [22]); (iv) obstetric history: gravidity, history of stillbirth; (v) pregnancy follow-up: number of prenatal visits (adequate follow-up defined as at least three prenatal visits at recruitment, according to WHO recommendations [23]), and intermittent preventive treatment of malaria in pregnancy with sulfadoxine-pyrimethamine (SP-IPTp) (defined as an intake of at least one dose during pregnancy).

### Samples analyses

After microscopic examination and Gram’s coloration, isolates were plated onto selective growth medium, Granada Medium (Becton Dickinson) for group B Streptococcus, and CHROMagarTM Candida for yeast isolation) and incubated for 24–48 h at 37 °C in 5% CO_2_. Vaginal flora were classified into four categories, based on microscopic examination and Gram’s coloration of the vaginal smear: “Type I” Döderlein flora, “Type II” majority of *Lactobacillus *spp. associated with few bacteria, “Type III” minority of *Lactobacillus *spp., “Type IV” no *Lactobacillus *spp. With knowledge of the microscopic examination and culture results, the bacteriologist could diagnose BV. In our study we defined BV as a “Type IV” vaginal flora.

Blood samples were screened for HBs antigen, *Toxoplasma gondii* and Rubella antibody immunoglobulin (Ig) G by enzyme immuno assay techniques (chimiluminescence Abbott Architect). All analyses were performed in the Biomedical Laboratory of the Pasteur institute in Dakar.

### Statistical methods

Continuous variables were expressed as median with interquartile range (IQR); discrete variables were expressed as percentage with 95% confidence interval (CI). BV-positive and BV-negative groups were compared using χ^2^ test or Fisher’s exact test for dichotomous variables and Student’s t-test or Wilcoxon rank-sum test for continuous variables.

All variables associated with BV in univariate analysis (p < 0.25) were then included in a backward stepwise logistic regression. Because rural/suburban setting was not associated with BV in univariate analysis (p > 0.25), it was not included in the final model. Interactions were assessed between age and gravidity. A p-value ≤ 0.05 was considered statistically significant. For univariate and multivariate analyses, age and gravidity were expressed as dichotomous variables using the median as the threshold for age (median age was 28), and primigravidity as the threshold for gravidity usually used in the literature [24, 25]). Data were analyzed using STATA Software Version 15.1 (Stata Corporation, College Station, Texas, USA).

### Ethics, data protection and confidentiality

The BIRDY protocol was approved by the relevant national ethics committees for health research of Senegal and France. Women were included after receiving information about the project, agreeing to providing biological samples, and signing an informed consent form. The BIRDY data collection has been declared to the *Commission Nationale de l’Informatique et des Libertés* (CNIL – French national data protection authority), in accordance with French law.

## Results

A total of 805 pregnant women were included in the Birdy study in Senegal. From October 2013 to September 2018, 477 (62%) women were screened for BV; 457 (96%) had no missing data for variable of interest (center, age, education, gravidity, number of prenatal consultations) and were analyzed, 308 (67.4%) in the suburban area and 149 (32.6%) in the rural area.

Sociodemographic characteristics, pregnancy follow-up, immune status and vaginal smear characteristics are described in Table 1.

**Table 1:** Characteristics of pregnant women, Senegal, 2013–2018

Characteristics		n/N	
Center	Suburban (Guédiawaye)	308/457	(67.4%)
	Rural (Sokone)	149/457	(32.6%)
Age (years)	Median (IQR)	28.1 (18.4–33.1)	
	< 20	50/457	(10.9%)
	20–24	94/457	(20.6%)
	25–29	138/457	(30.2%)
	30–35	99/457	(21.7%)
	> 35	76/457	(16.6%)
Marital status	Single	40/457	(8.8%)
	married	417/457	(91.2%)
Education	No formal education	164/457	(35.9%)
	Formal education^β^	293/457	(64.1%)
Sanitations	Improved ^μ^	229/456	(50.2)
	Unimproved	227/456	(49.8%)
Gravidity	Median (IQR)	3 (2–4)	
	1	117/457	(25.6%)
	2–4	256/457	(56.0%)
	≥ 5	94/457	(20.6%)
History of still birth	-	23/457	(4.9%)
Number of prenatal consultations	< 3	140/457	(30.6%)
	≥ 3	317/457	(69.4%)
SP-IPTp^α^	–	339/453	(74.8%)
Undernourished^Δ^	–	19/356	(5.3%)
Bacterial vaginosis	–	85/457	(18.6%)
Vaginal candidiasis	–	188/457	(41.1%)
Group B *Streptococcus* vaginal colonization	–	66/457	(14.5%)
HBs antigen positive	–	38/424	(9.0%)
*Toxoplasma gondii* antibody, IgG	Positive	159/449	(35.4%)
	Negative	277/449	(61.5%)
	Gray zone	13/449	(2.9%)
*Rubella* antibody, IgG	Positive	401/450	(89.1%)
	Negative	26/450	(5.8%)
	Gray zone	23/450	(5.1%)

^α^Intermittent preventive treatment of malaria in pregnancy with sulfadoxine-pyrimethamine

^Δ^Brachial perimeter < 24 cm use as a proxy of undernutrition

^β^Defined as at least primary education

^μ^Inside latrines and latrines with water flush were considered as improved sanitation facilities

### Population characteristics

Most women were married (417/457, 91.2%) and 293/457 (64.1%) had a formal education (at least primary education). Median age was 28.1 years (18.4–33.1). A total of 107/457 (23.4%) women were primigravida. Regarding pregnancy follow-up, most women attended at least three prenatal visits at enrollment (317/456, 69.4%); median number of prenatal visits was 2 (1–3), and a majority received at least one dose of SP-IPTp (339/453, 74.8%).

### Immune status

Among the 424 women tested for HBs antigen, 38 (9.0%) were positive. Whereas most were immunized against Rubella, 400/450 (89.1%), only about a third had IgG against *Toxoplasma gondii*, 159/449 (35.4%).

### Vaginal smear

Overall BV prevalence was 18.6% (85/457) [95% CI 15.4–22.6]) and was similar in suburban and rural areas (18.9% versus 18.1%, p = 0.843). Vaginal flora type on vaginal smear are described in Table 2 according to location (suburban/rural). A total of 102 (22.3%) women had *Gardnerella vaginalis* colonization. *Gardnerella vaginalis* was associated with BV in 73/457 vaginal smears (16.0%). Regarding other types of bacterial colonization, group B *Streptococcus*, and *Candida spp.* were found in 66/457 (14.5%) and 188/457 (41.1%), respectively.

**Table 2:** Vaginal flora type according to location in pregnant women, Senegal, 2013–2018

Vaginal Flora type^α^	Total	Suburban (Guédiawaye)			Rural (Sokone)		p-value^β^
	n = 457	n = 308			n = 149		
I	174	37.9%	117	37.8%	57	38.3%	< 0.001
II	63	13.8%	56	18.2%	7	4.7%	< 0.001
III	135	29.6%	77	25.1%	58	38.9%	< 0.001
IV*	85	18.6%	58	18.9%	27	18.1%	0.843

^α^Vaginal Flora Type defined as followed: “Type I” Döderlein flora, “Type II” majority of Lactobacillus spp. associated with few bacteria, “Type III” minority of Lactobacillus spp., “Type IV” no Lactobacillus spp.

*Defined as bacterial vaginosis in our study

^β^p-value comparing vaginal flora type between suburban and rural areas

### Factors associated with bacterial vaginosis (Table 3)

**Table 3:** Bacterial vaginosis (BV) risk factors in Senegalese pregnant women, 2013–2018

		BV + N = 85		BV – N = 371		Univariate analysis		Multivariate analysis	
		n	%	n	%	crude OR	CI 95%	Adjusted OR	CI 95%
Age (years)	< *28*	29	34.1	199	53.5	Ref	–		
	≥ *28*	56	65.9	173	46.5	2.22	1.35–3.66		
Location	Suburban (Guédiawaye)	58	68.2	249	67.1	Ref	–		
	Rural (Sokone)	27	31.8	122	32.9	0.95	0.57–1.57		
Education	Formal education^β^	36	42.4	128	34.5	Ref	–		
	No formal education	49	57.6	243	65.5	0.71	0.44–1.16		
Gravidity	Primigravida Multigravida Primigravida	9	10.6	98	26.4	Ref	–	Ref	-
	Multigravida	76	89.4	273	73.6	3.02	1.44–6.31	2.88	1.39–6.00
History of still birth	No	78	91.8	355	95.7		–		
	Yes	7	8.2	16	4.3	2.00	0.79–5.03		
Prenatal consultations	≥ *3*	19	77.7	121	67.4	Ref	–	Ref	–
	< *3*	66	22.3	250	32.6	1.67	0.96–2.92	1.55	0.88–2.70
Undernourished^*Δ*^	No	62	95.4	274	94.5	Ref	–		
	Yes	3	4.6	16	5.5	0.83	0.23–2.94		
SP-IPTp^α^	Yes	62	73.8	276	75.0	Ref	–		
	No	22	26.2	92	25.0	1.06	0.62–1.83		
Vaginal candidiasis	No	56	65.9	212	57.1	Ref	–		
	Yes	29	34.1	159	42.9	0.69	0.42–1.14		
Group B *Streptococcus* vaginal colonization	No	73	85.9	317	85.4	Ref	–		
	Yes	12	14.1	54	14.6	0.96	0.49–1.90		

^α^Intermittent preventive treatment of malaria in pregnancy with sulfadoxine-pyrimethamine

^Δ^Brachial perimeter < 24 cm use as a proxy of undernutrition

^β^Defined as at least primary education

In univariate analysis, being at least 28 years of age and multigravida was associated with a higher risk of BV, OR 2.22 [95% CI 1.35–3.66] and OR 3.02 [95% CI 1.44–6.31], respectively.

In multivariate analysis, multigravidity was the only factor to be found significantly associated with a higher risk of BV (aOR 2.88 [95% CI 1.39–6.00]).

Location, education status, nutritional status and history of stillbirth were not significantly associated with BV in univariate analysis, nor were pregnancy follow-up indicators (number of prenatal visits and SP-IPTp, Group B Streptococcus vaginal colonization, was not significantly associated with BV, OR 0.69 [95% CI 0.42–1.14].

## Discussion

To our knowledge, this is the first study of BV among Senegalese pregnant women. Our study shows that BV prevalence in pregnant women was 18.6%. This estimate is lower than that reported among non-pregnant Senegalese women (39.5%), in which BV detection was performed only in symptomatic women [19]. Our result is consistent with other studies carried out among pregnant women, regardless of the presence/absence of symptoms [4, 17, 18, 26].

We identified an association between gravidity and BV: multigravida women were more likely to have BV than nulligravida women. The role of gravidity in BV remains unclear. One Australian case–control study among 1780 women showed a similar association between multigravida women at higher risk for BV (OR 1.5, p < 0.0006) [24], whereas a Japanese study on 6083 women found no association between BV and gravidity in a multivariate analysis [25]. Sexual activity is a known risk factor of BV, as shown in a meta-analysis of 43 studies, which concluded that women with new or multiple male partners were 1.6 times more at risk of BV [27]. Primigravidity could reflect a lower level of sexual activity, particularly in Senegal where most women have no sexual partners before marriage and usually become pregnant in the first year after marriage.

A meta-analysis studying the association between vaginal microbiota and various STI found a protective role of high-*Lactobacillus* vaginal microbiota for HPV and *C. trachomatis* [28]. Almost half of the women included in our study had low-*Lactobacillus* vaginal microbiota (type III and IV vaginal flora), suggesting that they may be at higher risk of STI, including *C. trachomatis* infection, which is associated with adverse pregnancy outcomes [29].

We found no association between BV and GBS vaginal colonization. This association remains poorly investigated, and study results are inconsistent. In vitro studies showed that GBS adherence and biofilm formation are regulated by pH and thus by the abundance of *Lactobacillus* [30, 31]*.* Culture-based studies reported a positive association between GBS and low level of *Lactobacillus,* whereas a recent 16 s gene amplification study did not, but the latter focused on non-pregnant women [32]. As this study’s authors acknowledged, the abundance of *Lactobacillus* and alpha-diversity are modified during pregnancy. In addition, investigating interactions between GBS colonization and microbiota composition is hampered by the cross-sectional design of the studies.

Another interesting finding of this study is the low immunity rate against *Toxoplasma gondii*, i.e. 36.5%. A prior study conducted in Dakar among women of reproductive age found similar results (40.3% immunity rate) [33]. Seroprevalence of *Toxoplasma gondii IgG* varies from 50 to 80% in Arab and African countries [34, 35] and from 10 to 30% in European and North American countries [36–39]. *Toxoplasma gondii* transmission mainly occurs through the ingestion of bradyzoites in raw or undercooked meat products and through sporozoite ingestion from soil-contaminated fruit and raw vegetables. In Senegal, food preparations usually rely on overcooked meat and cooked vegetables, which may explain lower immunity against *Toxoplasma gondii.*

### Limitations

BV diagnosis routinely performed at the Pasteur Institute of Dakar differs slightly from the Amsel criteria, a clinical and microscopic approach, and from the Nugent criteria based on microscopic examination of vaginal swabs. The latter is used for research purposes because its inter-center reproducibility allows for more reliable comparisons. This procedure, however, requires more time, resources and expertise [40]. Sokone is a rural setting located more than 200 km from Dakar and could be considered as a resource limited setting. Moreover, in the Pasteur Institute of Dakar, microbiologists do not routinely perform the Nugent score, so that BV diagnosis is based on a microscopic examination and culture of vaginal swabs. We therefore used the vaginal flora type classification to define BV, a procedure carried out on a daily basis for years at the Pasteur Institute of Dakar, in order to limit inter and intra operator variability. In studies on BV prevalence in pregnant women [4, 17, 18, 26], the BV diagnosis method primarily relied on the Nugent score and Amsel criteria. Nonetheless, we find in our study that BV prevalence is consistent with that among in pregnant women in other studies.

## Conclusion

BV has long been well known, but few studies have reported BV prevalence among African pregnant women. No data for BV among pregnant women in Senegal exists, and women do not undergo screening for it as part of their antenatal screening. Although several studies showed that BV is associated with a higher risk of adverse obstetrics outcomes during pregnancy, the impact of BV on obstetrics outcomes and pregnancy in African countries has not been studied. Before authorities consider offering systematic BV screening to pregnant women, a larger study would be useful to document prevalence, risk factors and the impact of BV on adverse pregnancy outcome in sub-Saharan African countries.

## Data Availability

The BIRDY data collection has been declared to the Commission Nationale de l’Informatique et des Libertés (CNIL – French national data protection authority), in accordance with French law. The datasets used and/or analysed during the current study are available from the corresponding author on reasonable request.

## Abbreviations

aOR: Adjusted odd ratio
BV: Bacterial vaginosis
CI: Confidence interval
Ig: Immunoglobulin
IQR: Interquartile range
OR: Odd ratio
SP-IPTp: Intermittent preventive treatment of malaria in pregnancy with sulfadoxine-pyrimethamine
STI: Sexually transmitted infections
WHO: World Health Organization
